# Prevalence, trends, patterns and associations of analgesic use in Germany

**DOI:** 10.1186/s40360-015-0028-7

**Published:** 2015-10-01

**Authors:** Giselle Sarganas, Amanda K. Buttery, Wanli Zhuang, Ingrid-Katharina Wolf, Daniel Grams, Angelika Schaffrath Rosario, Christa Scheidt-Nave, Hildtraud Knopf

**Affiliations:** Robert Koch Institute, Department of Epidemiology and Health Monitoring, General-Pape-Str. 64, 12101 Berlin, Germany

**Keywords:** Analgesic use, Prevalence, Trends, Over-the-counter, Population based

## Abstract

**Background:**

Despite the public health relevance of analgesic use, large-scale studies on this topic in Germany are lacking. This study describes the prevalence, trends, associations and patterns of use of prescription and over-the-counter (OTC) analgesics, focusing on five of the most common agents: aspirin, diclofenac, ibuprofen, naproxen and paracetamol.

**Methods:**

Data from two representative population-based surveys: The German National Health Interview and Examination Survey 1998 (GNHIES98 *n* = 7099) and the German Health Interview and Examination Survey for Adults 2008–2011 (DEGS1 *n* = 7091) was investigated. Information on all medicines consumed in the previous 7 days was collected via computer-assisted personal interviews with adults aged 18–79 years. Associations between analgesic use and socio-demographic and health-behaviour factors were analysed using logistic regression models.

**Results:**

Analgesic use has increased over the last decade from 19 to 21 %. This was exclusively due to the rise in OTC analgesic use from 10.0 to 12.2 %. Prescribed analgesic use remained constant (7.9 %). Findings from DEGS1 indicate that ibuprofen is the most commonly used analgesic followed by aspirin and paracetamol. OTC analgesic use is higher among women and smokers, but lower among older adults (65–79 years). Prescribed analgesics use is higher among women, older adults, smokers and obese adults with medium or high socio- economic status. Adults performing more than 2 h/week of physical exercise use fewer analgesics.

**Discussion:**

Among the adult population of Germany, the prevalence of OTC analgesic use has significantly increased over the last decade. We found differences between adults consuming OTC and prescribed analgesics (or both) concerning their health behaviour and health conditions. International direct comparison between prevalence rates of analgesic use was limited due to varying availability of analgesics between countries and to methodological differences.

**Conclusions:**

About one in five community dwelling adults aged 18–79 years in Germany use analgesics in a given week. Considering the potential harms of analgesic use, monitoring of prevalence, patterns and determinants of use at the population level are important steps to inform disease prevention and health promotion policies.

## Background

Non-steroidal anti-inflammatory drugs (NSAIDs) and paracetamol (acetaminophen) are essential drugs to treat pain. The availability of medicines with analgesic effects varies extensively between countries [1]. Debates about the suitability of NSAIDs for over-the-counter (OTC) sale [2] versus prescription dispensing focus on the considerable health risks associated with their inappropriate use including drug intoxication, drug interactions and adverse drug reactions [3–6]. For example, the intake of diclofenac, ibuprofen and naproxen increase the risk of gastrointestinal complications by about 2–4 times and doubles the risk of heart failure requiring hospital admission [7]. Paracetamol, a common and easily accessible drug in many countries and included in approximately 150 preparations [8] carries risks of intoxication with 10 to 15 g reported as hepatotoxic for adults and 25 g can be life-threatening [8]. Strong relationships between paracetamol sales and non-fatal overdoses in England and France have been reported and rising sales are associated with increasing abuse resulting in liver damage, non-fatal self-poisonings and suicides [9, 10]. In the US, 458 people die per year because of paracetamol overdose and about 100 of these are accidental [11]. Since April 2009 in Germany OTC-sales of paracetamol are restricted to 10 g of active ingredients per package [12]. Drug regulatory authorities and healthcare systems face challenges in balancing the availability of analgesics in the market while controlling their associated risks, particularly in the context of ageing populations and rising long term conditions.

In Germany, in addition to prescription dispensing, aspirin, diclofenac, ibuprofen, naproxen and paracetamol are widely available OTC at pharmacies (not supermarkets) and through internet pharmacies, always involving professional pharmacists in the sale. According to the national pharmaceutical manufacturers’ association (‘Bundesverband der Arzneimittelhersteller e.V.’) people in Germany spent on average just over 60€ per person on all OTC preparations, including analgesics in 2012 [13].

Analgesic consumption is critically relevant for public health, but representative population-based data on their actual use is rare. Most of the existing analgesic use studies are based on prescription-, health care insurance-, or secondary care data. This study describes the prevalence of actual analgesics use (prescribed and self-medication) in a representative German sample of non-institutionalized adults aged 18–79 years using data from two German National Health Interview and Examination Surveys (1998 and 2008–2011) conducted by the Robert Koch Institute. We focus on five common analgesics available both OTC and with prescription in Germany [14]: aspirin, diclofenac, ibuprofen, paracetamol and naproxen. We describe prevalences of analgesic use, factors associated with their consumption, patterns of their use and differences over the decade between the two surveys.

## Methods

### Study population

The present study is based on data from two national health examination surveys conducted by the Robert Koch Institute (RKI): the German National Health Interview and Examination Survey 1998 (GNHIES98) and the German Health Interview and Examination Survey for Adults 2008–2011 (DEGS1) [15]. Participants were recruited by a nationwide two-stage clustering sample design in both surveys. In the first stage, communities, representative for size, location and structure of German communities were selected. In the second stage a representative sample of adults aged 18–79 years was drawn from local population registers of those communities (7124 GNHIES98 participants from 120 communities and 8152 DEGS1 participants from 180 communities). All GNHIES98 participants were invited to re-participate in DEGS1 (response rate 62 %, *n* = 3959). An additional cross-sectional sample of 4193 first-time participants (response rate 42 %) was recruited for DEGS1 based on the same sampling design, in order to achieve a nationally representative sample of the population 18–79 years of age. GNHIES98 and DEGS1 employ identical survey methods and measurement protocols (self-administered questionnaires, computer-assisted personal interviews (CAPI), medical examinations, physiological measurements and tests and the collection of blood and urine samples) enabling valid comparisons between the surveys [15, 16].

### Data collection

Current medication intake was recorded via CAPI in the local study centres. In the invitation letter, participants were requested to bring all original packages of medicines or dietary supplements they had used in the previous 7 days. Participants were asked: *“Have you taken any medicinal products or dietary supplements within the last 7 days, such as vitamins or minerals? Please don’t forget any painkillers, insulin preparations, medications issued by a physician, injections or plant-based medicinal products and please also list medications from the supermarket or drugstore”*.

In the GNHIES98 study, the name of the product, the Anatomical Therapeutic Chemical (ATC) code, the application form (e.g. oral or topical) and the standard package size were recorded by the interviewer manually. In DEGS1, the information was scanned directly from the package. Most data were collected from original packages brought by participants. Only 1.5 % of participants needed to be subsequently contacted by telephone or mail to complete the medicine information required.

Additionally, information on the origin of medicines was collected. This included whether medicines were i) prescribed by a physician; ii) prescribed by a registered complementary medicine specialist (Heilpraktiker); iii) were obtained OTC or from other sources (e.g. the home medicine cabinet). Prescribed analgesic use was defined as preparations prescribed by a physician or a registered complementary medicine specialist. OTC analgesic use was defined as analgesics purchased without prescription or non-prescribed analgesics available at home from the medicine cabinet. Those with missing data on the origin of analgesics were classified as “analgesics from unknown origin”. If medicines were used regularly (at least 4 days/week), duration of usage was also recorded.

### Definition and identification of analgesic use

This study focuses on five commonly used analgesics: aspirin, diclofenac, ibuprofen, paracetamol and naproxen. Participants were defined as “analgesic users” according to the therapeutic effects: “analgesic”, “antipyretic” and “anti-inflammatory” based on the ATC codes. For example, aspirin is indicated for multiple uses including cardiovascular disease prevention as well as an analgesic. Only ATC codes related to analgesic effects were included and ATC codes related to other indications were excluded. Therefore in this analysis we restricted the included ATC codes to 7 out of 12 possible ATC codes for aspirin (A01AD05, M01BA03, N02AA66, N02BA01, N02BA51, N02BA71 and R05XA02); 6 out of 7 ATC codes for diclofenac (M01AB05, M01AB55, M02AA15, N02AA65, S01BC03 and S01CC01); 5 out of 7 ATC codes for ibuprofen (D09AC04, G02CC01, M01AE01, M01AE14 and M02AA13); and 5 out of the 7 ATC codes for paracetamol (N02AA69, N02BE01, N02BE51, N02BE61 and R05XA01). For naproxen all 5 ATC codes (G02CC02, M01AE02, M01AE52, M01AE56 and M02AA12) were included.

### Socio-demographic and health variables

Based on a literature review, we investigated a range of participants’ characteristics for associations with analgesic use. Socio-demographic characteristics included: age; gender; size of municipality categorised as rural (<5000 inhabitants), small town (5000 to <20,000 inhabitants), medium-sized town (20,000 to <100,000 inhabitants) and city (>100,000 inhabitants); socio-economic status (SES) was measured as an aggregated index including income, education and occupation. Education were assessed according to the International Standard Classification of Educational Degrees (ISCED-97) and categorised as low, medium and high [17, 18].

Health behaviour and conditions investigated included: body mass index (BMI) classified as underweight (<18.5 kg/m^2^), normal (18.5– <25 kg/m^2^), overweight (25– <30 kg/m^2^) and obese: (>30 kg/m^2^); smoking status categorised as regular smoker (smoking daily), occasional smoker (smoking less than daily), ex-smoker (previously smoking) and never-smoker; physical activity measured using standardised questions [19] and alcohol consumption measured using the Alcohol Use Disorders Identification Test (AUDIT-C) [20]. Women and men with more than four and five points respectively on the 12-point scale were classified as high-risk alcohol drinkers [21].

### Statistical analysis

Only participants who completed the interview on medication usage were included in the analyses (GNHIES98: *n* = 7099; DEGS1: *n* = 7091). To examine the prevalence of analgesic use, both cross-sectional and trend analyses were conducted using a weighting factor which corrects for deviations in the sample from the population structure with regard to age, sex, region of residence, and nationality, as well as municipality size and educational level reflecting the German population as of 31th Dec 2010 [22]. Calculation of the weighting factor also included the re-participation probability of GNHIES98 participants, based on a logistic regression model. Descriptive statistics were used to examine characteristics of the study population. Prevalence of overall analgesic use was grouped by each of the five analgesics and by source of origin (OTC only, prescribed only and participants using both, OTC and prescribed analgesics). Analgesic users were then stratified by socio-demographic and health characteristics. Categorical variables were expressed as percentages and 95 % confidence intervals (95 % CI) were reported. To examine relationships between analgesic use and socio-demographic characteristics and health behaviour, unadjusted and adjusted odds ratios (OR) and 95 % CI were determined using bivariate and multivariable logistic regression models with analgesic use as the dependent variable. Interactions of sex, age, SES, BMI category, smoking and physical exercise were tested for statistical significance. The models were run separately for overall analgesic use, use of OTC analgesics only and use of prescribed analgesics only. The SPSS-20 Complex Samples method was used in the analysis to account for the weighting and the clustering due to the two-stage sampling procedure. Group differences were considered statistically significant for *p*-values less than 0.05.

### Ethical considerations

Participants provided written informed consent prior to interview and examination in both surveys. GNHIES98 and DEGS1 were approved by the Federal and State Commissioners for Data Protection and the Charité-Universitätsmedizin Berlin ethics committee approved DEGS1 as well (No. EA2/047/08).

## Results

### Prevalence of analgesic use

Prevalence of analgesic use in 1998 (GNHIES98) and 2008–11 (DEGS 1) are presented in Table 1. Overall, analgesic use increased from 19.2 % in 1998 to 21.4 % in 2008–11. This is exclusively because of the significant rise in OTC analgesic use only from 10.0 to 12.2 %. Moreover, OTC analgesics use only increased in all subgroups of the population over this period, with stronger and statistically significant increases among women, adults aged 30–64 years, adults with low and medium SES and adults living in rural areas. In contrast, the prevalence of prescribed analgesic use remained constant in both surveys (7.9 %); the only increase observed over this period was among adults 30–44 years.

**Table 1:** Trends in prevalence of analgesic use in Germany between German Health Interview and Examination Surveys GNHIES98 and DEGS1

Study subjects	Overall analgesic use		OTC analgesic use only		Prescription analgesic use only		OTC and prescription analgesic use	
GNHIES98 *n* = 7099	GNHIES98	DEGS1	GNHIES98	DEGS1	GNHIES98	DEGS1	GNHIES98	DEGS1
DEGS1 *n* = 7091	*n* = 1311^e^	*n* = 1490^e^	*n* = 696	*n* = 805	*n* = 524	*n* = 583	*n* = 36	*n* = 66
	Prevalence (95 % CI)	Prevalence (95 % CI)	Prevalence (95 % CI)	Prevalence (95 % CI)	Prevalence (95 % CI)	Prevalence (95 % CI)	Prevalence (95 % CI)	Prevalence (95 % CI)
Total	**19.2 (18.1–20.3)**	**21.4 (20.1–22.6)**	**10.0 (9.2–10.8)**	**12.2 (11.2–13.2)**	7.9 (7.1–8.7)	7.9 (7.1–8.7)	0.5 (0.4–0.8)	0.9 (0.6–1.2)
Gender								
Men	16.1 (14.7–17.6)	17.6 (16.0–19.3)	8.2 (7.2–9.3)	9.4 (8.2–10.8)	7.0 (6.0–8.1)	7.2 (6.1–8.4)	0.3 (0.1–0.5)	0.4 (0.2–0.8)
Women	**22.3 (20.7–23.9)**	**25.1 (23.4–27.0)**	**11.8 (10.6–13.1)**	**14.9 (13.5–16.5)**	8.8 (7.7–10.0)	8.5 (7.5–9.7)	0.8 (0.5–1.3)	1.3 (0.9–1.8)
Age groups, years								
18–29	17.7 (15.3–20.4)	19.5 (16.8–22.5)	13.2 (11.1–15.6)	14.8 (12.5–17.6)	2.9 (2.0–4.2)	3.2 (2.2–4.7)	**0.1 (0.0–0.5)**	**0.7 (0.3–1.8)**
30–44	**19.1 (17.3–21.2)**	**23.8 (21.2–26.7)**	**13.3 (11.7–15.1)**	**16.4 (14.2–18.9)**	**4.6 (3.7–5.8)**	**6.8 (5.3–8.8)**	0.4 (0.2–0.8)	0.3 (0.2–0.7)
45–64	19.4 (17.7–21.3)	21.6 (19.7–23.7)	**9.4 (8.1–10.8)**	**12.0 (10.5–13.7)**	8.4 (7.2–9.7)	8.3 (7.0–9.7)	1.0 (0.6–1.5)	0.8 (0.4–1.3)
65+	20.2 (17.5–23.2)	19.5 (17.3–22.0)	3.7 (2.6–5.3)	4.5 (3.5–5.8)	15.8 (13.3–18.5)	12.8 (10.9–15.0)	**0.4 (0.1–1.4)**	**1.9 (1.2–3.0)**
Socio-economic status								
Low	**19.3 (16.7–22.2)**	**24.2 (21.3–27.4)**	**7.1 (5.6–9.0)**	**11.4 (9.3–13.9)**	10.2 (8.2–12.6)	10.8 (8.9–13.2)	0.8 (0.3–1.8)	1.3 (0.7–2.6)
Medium	19.5 (18.1–21.0)	20.9 (19.4–22.5)	**10.1 (9.1–11.3)**	**12.0 (10.8–13.3)**	8.1 (7.2–9.2)	7.6 (6.7–8.7)	0.4 (0.2–0.7)	0.8 (0.5–1.2)
High	18.7 (16.5–21.1)	19.7 (17.3–22.4)	12.6 (10.7–14.6)	13.1 (11.1–15.4)	4.9 (3.8–6.4)	5.5 (4.2–7.1)	0.8 (0.4–1.5)	0.7 (0.4–1.2)
Municipality size								
Rural^a^	16.3 (14.4–18.5)	20.1 (17.5–23.1)	**7.8 (6.4–9.4)**	**10.9 (8.9–13.2)**	7.8 (6.4–9.5)	8.0 (6.3–10.2)	0.4 (0.2–1.0)	0.8 (0.4–1.7)
Small town^b^	16.8 (14.7–19.1)	19.5 (17.3–21.9)	7.7 (6.4–9.3)	9.9 (8.3–11.7)	7.4 (6.0–9.2)	8.3 (6.8–10.1)	0.3 (0.1–0.9)	1.0 (0.5–1.9)
Town^c^	20.3 (18.3–22.6)	22.0 (19.9–24.3)	10.9 (9.3–12.6)	13.2 (11.4–15.2)	8.3 (6.9–10.0)	7.2 (5.9–8.6)	0.5 (0.2–0.9)	1.2 (0.7–1.9)
City^d^	21.4 (19.4–23.6)	22.8 (20.5–25.4)	12.0 (10.4–13.7)	13.6 (11.7–15.8)	7.8 (6.5–9.4)	8.1 (6.7–9.8)	0.8 (0.5–1.5)	0.5 (0.3–0.9)

Bold text denotes statistical significance (*p* < 0.05) when comparing GNHIES98 with DEGS1

^a^ = <5000 inhabitants. ^b^ = 5000 to <20,000 inhabitants. ^c^ = 20,000 to <100,000 inhabitants. ^d^ = ≥100,000 inhabitants

^e^Analgesics of unknown origin in GNHIES98 (*n* = 55) and in DEGS1 (*n* = 36)

Prevalence and socio-demographic factors associated with specific analgesic use in 2008–11 are presented in Table 2. Overall, ibuprofen was the most commonly used analgesic. Naproxen was seldom used. Women used more ibuprofen and paracetamol than men. Paracetamol use decreased with age while diclofenac use increased with age. SES was associated mainly with diclofenac use; those with lower SES were more likely to use diclofenac and naproxen than other SES groups.

**Table 2:** Prevalence of specific analgesic use among the adult population in Germany. German Health Interview and Examination Survey for Adults (DEGS1) 2008–2011

Analgesic use	Aspirin *n* = 399	Diclofenac *n* = 352	Ibuprofen *n* = 352	Paracetamol *n* = 330	Naproxen *n* = 12
	Prevalence (95 % CI)	Prevalence (95 % CI)	Prevalence (95 % CI)	Prevalence (95 % CI)	Prevalence (95 % CI)
Total	5.8 (5.1–6.6)	4.4 (3.8–5.0)	8.0 (7.2–8.9)	5.2 (4.6–5.9)	0.2 (0.1–0.3)
Gender					
Men*	5.2 (4.3–6.2)	4.3 (3.5–5.2)	**5.7 (4.8–6.8)**	**4.0 (3.2–4.9)**	0.1 (0.0–0.4)
Women	6.3 (5.3–7.5)	4.4 (3.7–5.3)	**10.3 (9.1–11.6)**	**6.4 (5.4–7.6)**	0.2 (0.1–0.4)
Age groups, years					
18–29*	4.8 (3.3–6.9)	**1.2 (0.6–2.2)**	**8.8 (7.0–11.0)**	**6.9 (5.3–9.1)**	0.0 (0.0–0.0)
30–44	5.7 (4.2–7.6)	**2.8 (1.9–4.3)**	9.5 (7.8–11.4)	7.0 (5.6–8.7)	0.2 (0.1–0.7)
45–64	6.5 (5.3–7.8)	**4.7 (3.9–5.7)**	7.7 (6.4–9.1)	**4.5 (3.5–5.7)**	0.1 (0.0–0.3)
65+	5.4 (4.3–6.9)	**8.8 (7.1–10.7)**	**6.0 (4.7–7.7)**	**2.6 (1.7–4.1)**	0.4 (0.1–1.3)
Socio-economic status					
Low*	4.8 (3.3–6.8)	**6.5 (5.0–8.5)**	9.5 (7.5–12.1)	5.2 (3.6–7.3)	**0.4 (0.2–1.1)**
Medium	5.6 (4.7–6.7)	**3.9 (3.3–4.7)**	7.9 (7.0–9.0)	5.5 (4.6–6.5)	**0.1 (0.0–0.3)**
High	7.1 (5.8–8.7)	**3.6 (2.6–4.9)**	6.8 (5.3–8.6)	4.1 (3.0–5.7)	**0.0 (0.0–0.3)**
Municipality size					
Rural^a^*	5.1 (3.7–7.0)	5.8 (4.2–8.0)	7.1 (5.4–9.3)	3.8 (2.9–5.0)	0.1 (0.0–0.7)
Small town^b^	5.0 (4.0–6.3)	3.9 (2.9–5.3)	7.0 (5.7–8.6)	5.1 (4.0–6.6)	0.2 (0.1–0.6)
Town^c^	5.9 (4.8–7.2)	4.6 (3.7–5.8)	8.6 (7.3–10.2)	5.6 (4.5–7.0)	0.1 (0.0–0.4)
City^d^	6.6 (5.1–8.4)	3.7 (2.8–4.8)	8.7 (7.2–10.3)	5.6 (4.3–7.2)	0.2 (0.1–0.8)

Bold text denotes statistical significance (*p* < 0.05) in comparison with the reference (*)

^a^ = <5000 inhabitants. ^b^ = 5000 to <20,000 inhabitants. ^c^ = 20,000 to <100,000 inhabitants. ^d^ = ≥100,000 inhabitants

**Fig. 1:**
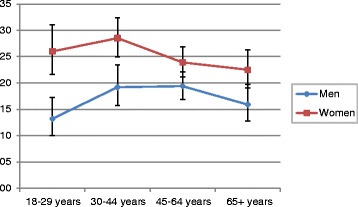
Prevalence of analgesic use among the adult population in Germany by age and gender; German Health Interview and Examination Survey for Adults (DEGS1) 2008–2011

In 2008–11, women’s use of analgesics was significantly higher than men’s (25.1 % vs. 17.6 %, OR = 1.58, 95 % CI 1.35–1.85). Figure 1 presents the prevalence of analgesic use in the last 7 days by age and gender. Analgesic consumption was lowest among men aged 18–29 years and highest among women aged 30–44 years.

Regardless of the source of origin (OTC or prescribed), 11.0 % (164/1490) of analgesic users consumed two different analgesics (of the five pharmaceutical active ingredients examined) during the 7 day period. Among these, 4.9 % (74/1490) used a combination of paracetamol with an NSAID, most frequently ibuprofen. 6.0 % of the participants (90/1490) report using two different NSAIDs during the 7-day period; these were predominately combinations of diclofenac and aspirin or ibuprofen. Only 0.9 % (66/1490) of analgesic users reported using both OTC and prescribed analgesics during the 7 days.

In 2008–11, 4.5 % of the population consumed analgesics regularly (at least 4 days/week). Of these, most reported using prescribed analgesics regularly (3.7 %; 95 % CI 3.2–4.3) and fewer reported regular OTC analgesic use (0.8 %; 95 % CI 0.6–1.1). Regarding the duration of use, 0.6 % of the population used OTC analgesics regularly and for more than 7 days and for prescribed analgesics the prevalence was higher (3.2 %).

### Associations of analgesic use with socio-demographic and health characteristics

Table 3 presents the bivariate logistic regression analyses and the associations between analgesics use and socio-demographic and health characteristics. Overall analgesic use was higher among women; adults aged 30–44 years, adults with obesity and smokers. Participants with high education levels and those performing more than 4 h of physical exercise per week consumed significantly less analgesics. Among all analgesic users (*n* = 1490), more participants reported consuming only OTC analgesics compared to only prescribed analgesics (12.2 % vs. 7.9 %). OTC use was higher among women and smokers, but lower among those aged 45 years and older and among overweight or obesity adults.

**Table 3:** Bivariate logistic regression analysis: prevalence and factors associated with analgesic use among the adult population in Germany. German Health Interview and Examination Survey for Adults (DEGS1) 2008–2011

	Study	Overall analgesic use *n* = 1490^b^		OTC analgesic use only *n* = 805		Prescription analgesic use only *n* = 583		OTC and prescription analgesic use *n* = 66	
	Subjects	Prevalence	Unadjusted	Prevalence	Unadjusted	Prevalence	Unadjusted	Prevalence	Unadjusted
	*n* (%)	(95 % CI)	OR (95 % CI)	(95 % CI)	OR (95 % CI)	(95 % CI)	OR (95 % CI)	(95 % CI)	OR (95 % CI)
Total	7091 (100)	21.4 (20.1–22.6)	-	12.2 (11.2–13.2)	-	7.9 (7.1–8.7)	-	0.9 (0.6–1.2)	-
Gender									
Men^a^	3399 (49.7)	17.6 (16.0–19.3)	1.00	9.4 (8.2–10.8)	1.00	7.2 (6.1–8.4)	1.00	0.4 (0.2–0.8)	1.00
Women	3692 (50.3)	**25.1 (23.4–27.0)**	**1.58 (1.35–1.85)**	**14.9 (13.5–16.5)**	**1.68 (1.36–2.07)**	8.5 (7.5–9.7)	1.21 (0.97–1.49)	**1.3 (0.9–1.8)**	**2.87 (1.39–5.94)**
Age groups, years									
18–29^a^	1065 (18.8)	19.5 (16.8–22.5)	1.00	14.8 (12.5–17.6)	1.00	3.2 (2.2–4.7)	1.00	0.7 (0.3–1.8)	1.00
30–44	1426 (25.1)	**23.8 (21.2–26.7)**	**1.29 (1.01–1.66)**	16.4 (14.2–18.9)	1.13 (0.85–1.49)	**6.8 (5.3–8.8)**	**2.19 (1.33–3.61)**	0.3 (0.2–0.7)	0.49 (0.14–1.76)
45–64	2750 (36.5)	21.6 (19.7–23.7)	1.14 (0.93–1.39)	**12.0 (10.5–13.7)**	**0.78 (0.62–0.98)**	**8.3 (7.0–9.7)**	**2.68 (1.74–4.13)**	0.8 (0.4–1.3)	1.11 (0.38–3.31)
65+	1850 (19.6)	19.5 (17.3–22.0)	0.99 (0.79–1.26)	**4.5 (3.5–5.8)**	**0.27 (0.19–0.37)**	**12.8 (10.9–15.0)**	**4.38 (2.78–6.89)**	1.9 (1.2–3.0)	2.79 (0.92–8.52)
Socio-economic status									
Low^a^	1156 (19.7)	24.2 (21.3–27.4)	1.00	11.4 (9.3–13.9)	1.00	10.8 (8.9–13.2)	1.00	1.3 (0.7–2.6)	1.00
Medium	4206 (60.1)	20.9 (19.4–22.5)	0.83 (0.67–1.02)	12.0 (10.8–13.3)	1.07 (0.81–1.40)	**7.6 (6.7–8.7)**	**0.68 (0.52–0.89)**	0.8 (0.5–1.2)	0.58 (0.27–1.26)
High	1685 (20.2)	**19.7 (17.3–22.4)**	**0.77 (0.61–0.98)**	13.1 (11.1–15.4)	1.17 (0.87–1.58)	**5.5 (4.2–7.1)**	**0.48 (0.33–0.68)**	0.7 (0.4–1.2)	0.52 (0.21–1.32)
Education status (ISCED)									
Low	1003 (21.1)	20.3 (17.6–23.3)	0.87 (0.71–1.05)	10.6 (8.5–13.2)	0.80 (0.61–1.05)	8.2 (6.7–10.1)	0.97 (0.74–1.28)	1.2 (0.6–2.2)	1.39 (0.63–3.04)
Medium^a^	3773 (55.1)	22.8 (21.2–24.4)	1.00	12.9 (11.6–14.3)	1.00	8.4 (7.4–9.6)	1.00	0.9 (0.6–1.3)	1.00
High	2271 (23.7)	**18.7 (16.6–20.9)**	**0.78 (0.66–0.92)**	11.6 (9.8–13.8)	0.89 (0.70–1.13)	**6.0 (4.9–7.4)**	**0.69 (0.54–0.91)**	0.6 (0.4–1.0)	0.69 (0.37–1.32)
Body Mass Index (BMI)									
Underweight (<18.5)	93 (1.5)	26.5 (16.6–39.5)	1.77 (0.79–2.64)	22.1 (13.0–35.2)	1.79 (0.93–3.44)	4.4 (0.9–18.1)	0.80 (0.16–3.95)	0.0 (0.0–0.0)	0.0 (0.0–0.0)
Normal (18.5–< 25)^a^	2599 (38.4)	20.0 (18.3–21.9)	1.00	13.7 (12.3–15.3)	1.00	5.4 (4.4–6.6)	1.00	0.3 (0.2–0.7)	1.00
Overweight (25–< 30)	2651 (36.4)	20.5 (18.4–22.7)	1.03 (0.86–1.23)	**11.2 (9.6–13.1)**	**0.79 (0.64–0.99)**	**8.2 (6.9–9.7)**	**1.57 (1.17–2.09)**	**0.8 (0.5–1.2)**	**2.57 (1.08–6.14)**
Obese (≥30)	1698 (23.6)	**24.7 (22.1–27.5)**	**1.31 (1.09–1.57)**	**10.7 (9.0–12.6)**	**0.75 (0.61–0.93)**	**11.6 (9.9–13.7)**	**2.31 (1.75–3.06)**	**1.9 (1.2–3.0)**	**5.97 (2.52–14.2)**
Smoking status									
Never-smoker^a^	3067 (42.0)	19.1 (17.5–20.9)	1.00	10.8 (9.4–12.3)	1.00	7.0 (5.9–8.2)	1.00	0.9 (0.5–1.4)	1.00
Ex-smoker	2111 (28.1)	20.2 (17.9–22.7)	1.07 (0.88–1.29)	10.1 (8.5–12.0)	1.65 (1.32–2.07)	8.3 (6.8–10.2)	1.21 (0.89–1.63)	1.2 (0.7–2.0)	0.67 (0.23–1.95)
Occasional-smoker	415 (6.1)	20.7 (16.4–25.8)	1.10 (0.81–1.50)	13.7 (10.1–18.3)	1.31 (0.89–1.92)	6.5 (4.0–10.5)	0.93 (0.53–1.61)	0.5 (0.2–1.6)	0.59 (0.17–2.08)
Smoker	1460 (23.7)	**26.6 (23.9–29.5)**	**1.53 (1.28–1.83)**	**16.6 (14.4–19.0)**	**1.65 (1.32–2.07)**	9.0 (7.3–10.9)	1.31 (0.99–1.72)	0.6 (0.2–1.4)	1.38 (0.64–2.96)
Physical exercise hours/week (h/w)									
None	2210 (33.1)	22.5 (20.5–24.6)	1.00 (0.85–1.18)	12.1 (10.3–14.1)	0.94 (0.74–1.19)	9.0 (7.7–10.5)	1.08 (0.86–1.37)	0.9 (0.6–1.4)	1.06 (0.55–2.02)
Up to 2 h/w^a^	2952 (41.4)	22.5 (20.6–24.4)	1.00	12.8 (11.2–14.5)	1.00	8.4 (7.2–9.7)	1.00	0.9 (0.6–1.4)	1.00
2–4 h/w	1078 (15.2)	19.0 (16.4–21.9)	0.81 (0.65–1.00)	13.3 (11.2–15.6)	1.04 (0.82–1.34)	**4.7 (3.2–6.9)**	**0.54 (0.36–0.83)**	0.3 (0.1–0.8)	0.35 (0.12–0.99)
More than 4 h/w	657 (10.3)	**17.2 (13.9–21–2)**	**0.72 (0.54–0.95)**	11.0 (8.3–14.5)	0.84 (0.59–1.19)	**5.1 (3.5–7.5)**	**0.59 (0.38–0.92)**	0.8 (0.2–2.8)	0.85 (0.21–3.45)
High risk alcohol drinking									
No^a^	4571 (66.6)	21.3 (20.0–22.8)	1.00	11.8 (10.7–13.0)	1.00	8.3 (7.4–9.3)	1.00	0.7 (0.5–1.1)	1.00
Yes	2252 (33.4)	21.7 (19.5–24.1)	1.02 (0.87–1.19)	13.7 (11.9–15.8)	1.19 (0.98–1.44)	6.7 (5.4–8.3)	0.79 (0.62–1.03)	0.9 (0.5–1.6)	1.23 (0.63–2.42)

Odds ratio (OR) and its 95 % confidence intervals (95 % CI) are obtained from bivariate analysis. Bold text denotes statistical significance (*p* < 0.05) in comparison with the reference

^a^Reference group. All percentages are weighted according to the adult German population

^b^Analgesics of unknown origin (*n* = 36)

Among participants reporting only taking prescribed analgesics, we found a strong association between increased age and the use of prescribed analgesics (Table 3). Adults with low SES use significantly more prescribed analgesics than adults with high SES. Higher education status is associated with less prescribed analgesic use. Being overweight or obese is associated with higher levels of prescribed analgesics. Performing 2 or more hours of physical activity per week was associated with lower prescribed analgesic use (Table 3).

Among all analgesic users (*n* = 1490), a total of 66 adults reported using both OTC and prescribed analgesics during the 7 days prior to the medical interview, demonstrating a weighted prevalence of 0.9 % (95 % CI 0.6–1.2 %). Significantly more women use both, OTC and prescribed analgesics in comparison to men.

Table 4 presents the multivariable logistic regression analysis. This analysis included socio-demographic variables (sex, age and SES) and health behavioral factors that can influence the use of analgesic such as physical exercise, smoking and BMI. Women were more likely to consume analgesics (OR = 1.63, 95 % CI 1.38–1.92) as were those currently smoking (OR = 1.55, 95 % CI 1.28–1.88) and those with obesity (OR = 1.33, 95 % CI 1.09–1.62). Adults 65 years and older were more likely to use only prescribed analgesics (OR = 3.64, 95 % CI 2.28–5.82) and less likely to use OTC analgesics (OR = 0.31, 95 % CI 0.22–0.44) compared to all other age groups. Adults with medium or high SES were less likely to use only prescribed analgesics. Obese adults were more likely to consume only prescribed analgesics (OR = 1.60, 95 % CI 1.18–2.19). Adults performing physical exercise for more than 2 h a week were less likely to consume only prescribed analgesics (OR = 0.71, 95 % CI 0.51–0.99) compared to adults performing no physical exercise.

**Table 4:** Multivariable logistic regression analysis: prevalence and factors associated with analgesic use, exclusively using OTC analgesics and exclusively using prescribed analgesics among the adult population in Germany German Health Interview and Examination Survey for Adults (DEGS1) 2008–2011

	Study	Analgesic use *n* = 1490^b^		OTC analgesic use only *n* = 805		Prescribed analgesic use only *n* = 583	
	Subjects	Prevalence	Adjusted	Prevalence	Adjusted	Prevalence	Adjusted
	*n* (%)	(95 % CI)	OR (95 % CI)	(95 % CI)	OR (95 % CI)	(95 % CI)	OR (95 % CI)
Total	7091 (100)	21.4 (20.1–22.6)	-	12.2 (11.2–13.2)	-	7.9 (7.1–8.7)	-
Gender							
Men^a^	3399 (49.7)	17.6 (16.0–19.3)	1.00	9.4 (8.2–10.8)	1.00	7.2 (6.1–8.4)	1.00
Women	3692 (50.3)	25.1 (23.4–27.0)	**1.63 (1.38–1.92)**	14.9 (13.5–16.5)	**1.71 (1.37–2.14)**	8.5 (7.5–9.7)	**1.28 (1.02–1.62)**
Age groups, years							
18–29^a^	1065 (18.8)	19.5 (16.8–22.5)	1.00	14.8 (12.5–17.6)	1.00	3.2 (2.2–4.7)	1.00
30–44	1426 (25.1)	23.8 (21.2–26.7)	1.27 (0.98–1.64)	16.4 (14.2–18.9)	1.16 (0.87–1.54)	6.8 (5.3–8.8)	**1.98 (1.19–3.28)**
45–64	2750 (36.5)	21.6 (19.7–23.7)	1.05 (0.84–1.33)	12.0 (10.5–13.7)	0.80 (0.61–1.05)	8.3 (7.0–9.7)	**2.19 (1.41–3.43)**
65+	1850 (19.6)	19.5 (17.3–22.0)	0.95 (0.73–1.23)	4.5 (3.5–5.8)	**0.31 (0.22–0.44)**	12.8 (10.9–15.0)	**3.64 (2.28–5.82)**
Socio-economic status							
Low^a^	1156 (19.7)	24.2 (21.3–27.4)	1.00	11.4 (9.3–13.9)	1.00	10.8 (8.9–13.2)	1.00
Medium	4206 (60.1)	20.9 (19.4–22.5)	0.85 (0.68–1.07)	12.0 (10.8–13.3)	1.03 (0.77–1.38)	7.6 (6.7–8.7)	**0.72 (0.54–0.95)**
High	1685 (20.2)	19.7 (17.3–22.4)	0.87 (0.66–1.15)	13.1 (11.1–15.4)	1.13 (0.81–1.58)	5.5 (4.2–7.1)	**0.62 (0.41–0.92)**
Body Mass Index (BMI)							
Normal (18.5–< 25)^a^	2599 (38.4)	20.0 (18.3–21.9)	1.00	13.7 (12.3–15.3)	1.00	5.4 (4.4–6.6)	1.00
Overweight (25–< 30)	2651 (36.4)	20.5 (18.5–22.8)	1.08 (0.90–1.30)	11.2 (9.6–13.1)	0.97 (0.77–1.23)	8.2 (6.9–9.7)	1.27 (0.94–1.71)
Obese (≥30)	1698 (23.6)	24.8 (22.2–27.6)	**1.33 (1.09–1.62)**	10.7 (9.0–12.6)	1.02 (0.81–1.29)	11.6 (9.9–13.7)	**1.60 (1.18–2.19)**
Smoking status							
Never-smoker^a^	3067 (42.0)	19.1 (17.5–20.9)	1.00	10.8 (9.4–12.3)	1.00	7.0 (5.9–8.2)	1.00
Ex-smoker	2111 (28.1)	20.2 (17.9–22.7)	1.16 (0.95–1.41)	10.1 (8.5–12.0)	1.09 (0.84–1.41)	8.3 (6.8–10.2)	1.17 (0.85–1.62)
Occasional smoker	415 (6.1)	20.7 (16.4–25.8)	1.16 (0.85–1.57)	13.7 (10.1–18.3)	1.20 (0.82–1.75)	6.5 (4.0–10.5)	1.16 (0.72–1.89)
Smoker	1460 (23.7)	26.6 (23.9–29.5)	**1.55 (1.28–1.88)**	16.6 (14.4–19.0)	**1.55 (1.22–1.95)**	9.0 (7.3–10.9)	**1.53 (1.10–2.13)**
Physical exercise hours/week (h/w)							
None^a^	2210 (33.1)	22.5 (20.5–24.6)	1.00	12.1 (10.3–14.1)	1.00	9.0 (7.7–10.5)	1.00
Up to 2 h/w	2952 (41.4)	22.5 (20.6–24.5)	1.08 (0.91–1.27)	12.8 (11.2–14.5)	1.08 (0.86–1.36)	8.4 (7.2–9.7)	1.08 (0.85–1.38)
More than 2 h/w	1735	18.3 (16.2–20.7)	0.91 (0.74–1.13)	12.3 (10.5–14.4)	1.08 (0.81–1.42)	4.9 (3.8–6.3)	**0.71 (0.51–0.99)**

Odds ratio (OR) and its 95 % confidence intervals (95 % CI) are obtained from multivariable logistic regression models with analgesic use, only OTC analgesic use or only prescribed analgesic use, respectively as dependent variable and all other variables shown in the table as independent variables. Bold text denotes statistical significance (*p* < 0.05) in comparison with the reference

^a^Reference group. All percentages are weighted according to the adult German population

^b^Analgesics of unknown origin (*n* = 36)

Table 5 presents the multivariable analysis including tests for interaction. Significant interaction terms were only found when prescription analgesic use only was the dependent variable (Table 5). There was a significant interaction between SES and BMI, where the association between obesity and a more frequent use of prescribed analgesics was not present among participants with a low SES. Likewise, the association between higher SES and a lower rate of prescribed analgesics use only was not present among obese adults. Results regarding the other factors were unaltered compared to Table 4.

**Table 5:** Multivariable logistic regression and interaction analysis: prevalence and factors associated with prescribed analgesic use among the adult population in Germany. German Health Interview and Examination Survey for Adults (DEGS1)^b^ 2008–2011

	Prescribed analgesic use only *n* = 583	
	Prevalence	Adjusted
	(95 % CI)	OR (95 % CI)
Total	7.9 (7.1–8.7)	-
Gender		
Men^a^	7.2 (6.1–8.4)	1.00
Women	8.5 (7.5–9.7)	**1.30 (1.04–1.64)**
Age groups, years		
18–29^a^	3.2 (2.2–4.7)	1.00
30–44	6.8 (5.3–8.8)	**2.04 (1.22–3.40)**
45–64	8.3 (7.0–9.7)	**2.21 (1.42–3.47)**
65+	12.8 (10.9–15.0)	**3.67 (2.29–5.87)**
Smoking status		
Never smoker^a^	7.0 (5.9–8.2)	1.00
Ex-smoker	8.3 (6.8–10.2)	1.17 (0.84–1.62)
Occasional smoker	6.5 (4.0–10.5)	1.20 (0.73–1.95)
Smoker	9.0 (7.3–10.9)	**1.52 (1.09–2.11)**
Physical exercise hours/week (h/w)		
None^a^	9.0 (7.7–10.5)	1.00
Up to 2 h/w	8.4 (7.2–9.7)	1.08 (0.85–1.38)
More than 2 h/w	4.9 (3.8–6.3)	**0.72 (0.52–0.99)**
Body Mass Index (BMI) by socio-economic status		
Low SES: Normal	7.6 (5.1–11.1)	0.87 (0.45–1.67)
Low SES: Overweight	14.0 (10.1–19.1)	1.44 (0.81–2.56)
Low SES: Obese^a^	11.0 (7.6–15.8)	1.00
Medium SES: Normal	5.6 (4.3–7.2)	**0.64 (0.44–0.92)**
Medium SES: Overweight	7.7 (6.1–9.6)	0.71 (0.50–1.02)
Medium SES: Obese^a^	11.1 (8.8–13.8)	1.00
High SES: Normal	3.6 (2.2–6.0)	**0.28 (0.14–0.56)**
High SES: Overweight	5.2 (3.5–7.7)	**0.37 (0.18–0.75)**
High SES: Obese^a^	13.9 (8.7–21.5)	1.00
Socio-economic status by Body Mass Index (BMI)		
Normal: Low SES	7.6 (5.1–11.1)	**2.07 (1.03–4.16)**
Normal: Medium SES	5.6 (4.3–7.2)	1.52 (0.82–2.83)
Normal: High SES^a^	3.6 (2.2–6.0)	1.00
Overweight: Low SES	14.0 (10.1–19.1)	**2.60 (1.42–4.78)**
Overweight: Medium SES	7.7 (6.1–9.6)	1.29 (0.79–2.12)
Overweight: High SES^a^	5.2 (3.5–7.7)	1.00
Obese: Low SES	11.0 (7.6–15.8)	0.67 (0.33–1.34)
Obese: Medium SES	11.1 (8.8–13.8)	0.67 (0.37–1.21)
Obese: High SES^a^	13.9 (8.7–21.5)	1.00

Odds ratio (OR) and its 95 % confidence intervals (95 % CI) are obtained from multivariable logistic regression models with exclusively prescribed analgesic use, as dependent variable and all other variables shown in the table as independent variables. Bold text denotes statistical significance (*p* < 0.05) in comparison with the reference

^a^Reference group. All percentages are weighted according to the adult German population

^b^Analgesics of unknown origin (*n* = 36)

Regarding factors associated with regular analgesic use, we did not find any socio-demographic nor health related factor associated with the regular OTC-analgesic use. However, factors positively associated with the regular prescribed analgesic use were: being male, older age, low SES, obesity and smoking.

## Discussion

Among the adult population of Germany, the prevalence of analgesic use has increased over the last decade. This rise is exclusively due to an increased in the OTC analgesic use, which was most notable among women and those with low SES. Furthermore, there are differences between adults consuming OTC and prescribed analgesics (or both) concerning their health behaviour and health conditions.

In Germany about one fifth of adults use analgesics in a given week; 12 % use exclusively OTC analgesics and 8 % use prescription analgesics exclusively. International direct comparison between prevalence rates of analgesic use is limited due to varying availability of analgesics between countries and to methodological differences, in particular, in the period of analgesic use investigated between studies. Nevertheless, our findings are comparable to the Slone Survey, a US telephone survey investigating medicines and supplements use in the previous 7 days, reporting that prescription and OTC analgesics are the most frequently used medical products among adults and are consumed by 17 to 23 % of the population [23]. Similarly, a contemporary study in France using national health insurance claims data investigating the usage patterns of OTC versus prescription NSAIDs (ibuprofen, diclofenac, naproxen and ketoprofen) reported that about 20 % of people receive at least one reimbursement of an OTC or prescription NSAID per year [24]. Similarly, an investigation of the patterns of NSAIDs use, based on population-level survey data from Denmark between 1997 and 2005, found that 15–17 % of people receive at least one prescription of NSAIDs (ibuprofen, diclofenac, naproxen, rofecoxib, celecoxib) per year [25]. However, prescription data from health insurance system cannot reflect the actual use of analgesics in the population as packages could be taken only partly and not all studies include private prescriptions and OTC self-medication. Furthermore, with prescription data it is not possible to estimate OTC use. Our survey data and the robust data collection methods we used to collect details about medicines taken in the previous 7 days may be more likely to reflect actual medicines consumed and not just prescribed.

In our study, OTC analgesic use significantly increased from 10 to 12 % within a decade. There are contextual trends that may explain this rise, e.g. as more analgesics move from prescription to OTC status, possibly leading to an increase of OTC analgesic use [26]. Availability, convenience, costs and product marketing are likely to impact on OTC analgesics use and self-medication in general. For example, an individual may consider the availability of local pharmacies, the convenience of attending their physician and costs associated with travel. Purchasing costs, including free of charge prescription medication may differ for individuals and are interrelated with personal circumstances and health insurance coverage. Rises in OTC analgesic use may be related to rising painful musculoskeletal chronic conditions, such as arthritis [27].

We found that the most commonly used analgesic in Germany is ibuprofen, followed by aspirin, paracetamol, diclofenac and naproxen. Higher frequency of use of ibuprofen compared to diclofenac has been reported in numerous countries including Denmark [28], the US [29] and in a comparative study of analgesic consumption in Slovakia, Norway and Finland [30]. An Australian study investigating patterns of OTC analgesic use between 2001 and 2009 finds a 15 % increase in the use of ibuprofen, naproxen, and diclofenac, whereas the relative use of paracetamol and aspirin declines by 14 and 4 % respectively, but an overall rise in the use of OTC NSAIDs is observed [31]. Conversely, there is evidence that OTC and prescribed diclofenac is more frequently used than ibuprofen in Serbia [32]. Furthermore, the afore cited Serbian study investigates trends over time for 2 years (2004–2006) and reports that, although there is an overall decline in the use of prescribed and OTC NSAIDs, OTC diclofenac and ibuprofen use increases over this period [32].

Naproxen is used comparatively rarely in Germany despite evidence that naproxen is associated with less vascular risk than other NSAIDs including ibuprofen and diclofenac [7]. Guidelines recommend the use of naproxen as the NSAID of choice for those at risk of cardiovascular complications [33, 34]. Low rates of naproxen use and prescribers and individuals preferences for certain NSAIDs and analgesics are influenced by a variety of factors, including the timing of introduction of an NSAID to the market [33] and pricing. Similar to other studies [2, 31], we find that 6 % (90/1490) of analgesic users consumed two different types of NSAIDs within the previous week. Using certain types of NSAID analgesics simultaneously at certain dosages and within specified time periods is contra-indicated and bears a high risk of adverse health events, such as gastric bleeding [35, 36] and myocardial infarction (MI) [37]. In our study, the proportion of adults, who uses regularly and for more than 7 days analgesics is relatively low (0.6 %). However, regular and long-term use of analgesics is linked to health risks [38] and further studies investigating those taking long term analgesics are required to understand the reasons for their use.

People aged 65 years or older as well as those with low SES use more diclofenac compared to other age groups. The vascular risks of high-dose diclofenac [7], particularly the risk of MI and death [25] are well established. Safety advice issued for diclofenac by the European Safety Agency, outlines how the drug is contra-indicated for people with established chronic heart failure, ischaemic heart disease, peripheral artery disease or cerebrovascular disease [39]. Older aged adults and people with low SES are more likely to suffer from cardiovascular disease [40], and our findings of increased diclofenac use among these groups warrants further investigation and explanation.

We found in our study that the rise in analgesic use over the past decade occurred mainly among those aged 30–44 years consistent with others reporting that younger people use more OTC analgesics than older people and that older people use more prescribed analgesics than younger people [31, 41]. We found women consume more analgesics than men, particularly more ibuprofen and paracetamol, consistent with others [31, 41–44]. There is evidence of a social gradient in analgesic use in Germany, in line with other studies conducted in European countries [2, 45]. People with low SES take more prescribed analgesics compared to those with high SES and high educational levels.

In our study certain health-behaviour factors were related to analgesics use. Similar to other studies, smoking was associated with a high prevalence of analgesic use [41, 46, 47]. This could be due to the fact that smoking is strongly associated with having more pain [48, 49]. We also observed a significant association between obesity and consuming more prescribed analgesics. Toblin et al. [50] have found that obesity was a risk factor for pain. Furthermore, in a Swedish study, being overweight was associated with more prescribed analgesic use among men and with more OTC analgesic use among women [41]. The association between obesity with activity difficulty because of pain was previously studied [51]. In our study, adults performing physical exercise for more than 4 h per week used fewer analgesics. Another study reported associations between higher exercise activities and more OTC consumption compared to prescribed analgesic use [52].

One of the main strengths of our study is the large nationally representative sample of non-institutionalised adults in Germany [22]. However, we recognise that response rates among newly recruited study participants in DEGS1 are lower than response rates of former GNHIES98 participants (42 % versus 62 %) [15]. We attempted to minimise response bias by applying corrective weights in our analysis. In both national surveys, GNHIES98 and DEGS1, people with severe diseases and impairments are likely to be under-represented and this analysis had an upper age limit of inclusion of 79 years. This could possibly have resulted in an underestimation of analgesic use.

In GNHIES98 and DEGS1 information on drug use was collected directly from participants and both, prescribed and OTC analgesic use was recorded. Collecting self-reported information on the use of medicines has strengths and limitations. Robust data collection methods using standardised CAPIs and scanning of original medicines packaging at the study centre improves the validation of self-reported medicines use [53]. By scanning the central pharmaceutical number, all information on the preparation, such as the name and ATC code, are transferred automatically to the database reducing the likelihood of transcription error. Restricting data collection in the survey to drug use during the last 7-days attempts to minimise errors due to poor memory, however the possibility of recall bias cannot be excluded. Furthermore, medicines used cyclically within and out of this 7-day time period could lead to misclassification and may potentially overestimate or underestimate the use of certain medicines.

## Conclusions

Based on nationally representative data the present study found that about one in five adults in Germany utilizes analgesics in a typical week. Rising use of analgesics over time is predominantly explained by increasing analgesics use among those aged 30–44 years and by rising OTC analgesic use, particularly among women and those with low SES. Levels of prescribed analgesic use in Germany remain the same as a decade ago. Ibuprofen is the most commonly used analgesic and women use more ibuprofen and paracetamol than men. Significant relationships exist between analgesics use and factors including age, gender, SES and health behaviour. Considering the potential harms of prolonged analgesic use, patterns of use at the population level need to be monitored in more detail. Further studies on high risk groups, those with co-morbidities, and those consuming multiple medicines are needed. Furthermore, patterns of analgesic use need to be examined in the context of pain management practice. Health policy planners and health workers face challenges with rising analgesics use and closer monitoring of adverse events and ways to minimise their risks are needed.

## Abbreviations

ATC: Anatomical Therapeutic Chemical
AUDIT-C: Alcohol Use Disorders Identification Test
BMI: Body mass index
CAPI: Computer-assisted personal interviews
DEGS1: German Health Interview and Examination Survey for Adults 2008–2011
GNHIES98: German National Health Interview and Examination Survey 1998
ISCED-97: International Standard Classification of Educational Degrees
NSAIDs: Non-steroidal anti-inflammatory drugs
OR: Odds ratio
OTC: Over-the-counter
RKI: Robert Koch Institute
SES: Socio-economic status
